# Recurrent meningoencephalitis and hyponatremia in childhood neurosarcoidosis

**DOI:** 10.1186/1546-0096-10-S1-A98

**Published:** 2012-07-13

**Authors:** Josephine Isgro, Shannon Babineau, Rachelle Gandica, Amy J Starr, Lisa F Imundo, Andrew H Eichenfield

**Affiliations:** 1 Morgan Stanley Children's Hospital of New York Presbyterian Hospital, Columbia University Medical Center, New York, NY, USA

## Purpose

Sarcoidosis is a rare multi-system inflammatory disease of unknown etiology, the pathology of which is characterized by the presence of non-caseating granulomas. Neurosarcoidosis can affect any part of the peripheral or central nervous system and occurs in approximately 5-15% of all sarcoidosis patients. In this report, we present a case of isolated neurosarcoidosis in a child manifested as recurrent meningoencephalitis and hyponatremia.

## Results

Case Report: A 13-year-old African American female with a past medical history significant for poorly controlled type 1 diabetes mellitus presented in July 2009 with altered mental status, headache, fever, and aseptic meningitis. MRI/MRA brain demonstrated punctate infarcts within deep white matter of frontal and parietal lobes as well as irregularity of left M2 and M3 branches and right M2 representing stenosis suspicious for vasculitis. Complete infectious disease workup, including tuberculosis, was negative. Rheumatologic evaluation was significant for a positive ANA (1:160), normal angiotensin converting enzyme, and elevated serum lysozyme at 30 ug/ml (range: 9-17). She developed hyponatremia (lowest 124 mM/l), which was attributed to cerebral salt wasting in the setting of meningitis, and treatment included oral sodium chloride and fludrocortisone, which resulted in normalization of her serum sodium. She made a complete and spontaneous recovery over the next week and was discharged with the presumptive diagnosis of viral encephalitis. One month after being discharged home, she neglected to refill her prescriptions for both the sodium chloride and fludrocortisone and her serum sodium remained within normal limits. Sixteen months later, with persistently poor control of her diabetes, she returned after the sudden onset of right-sided headache, altered mental status, waxing and waning left hemineglect and hemiplegia, and right hemispheric slowing on EEG. She was again noted to have hyponatremia, elevated serum lysozyme (21ug/ml), and CSF pleiocytosis with increased IgG synthetic rate and oligoclonal bands. Repeat MRI brain demonstrated slightly prominent right-sided leptomeningeal enhancement. She was restarted on oral sodium chloride supplementation and fludrocortisone. Slit lamp examination disclosed unilateral anterior uveitis with keratic precipitates. Posterior cervical lymph node biopsy demonstrated non-caseating granulomas consistent with sarcoidosis. Her symptoms resolved over the course of one week. However, two weeks following discharge, her headache returned and she subsequently suffered a third episode of encephalopathy for which she received high-dose intravenous corticosteroid with rapid and marked clinical improvement. Currently, she remains on treatment for cerebral salt wasting, without any neurologic deficits, on tapering doses of corticosteroids.

## Conclusion

Childhood neurosarcoidosis is a rare, pleiomorphic condition often posing a diagnostic challenge resulting in delayed recognition. Here we describe an unusual presentation of central nervous system involvement manifested by discrete and transient episodes of encephalomyelitis and hyponatremia from cerebral salt wasting.

## Disclosure

Josephine Isgro: None; Shannon Babineau: None; Rachelle Gandica: None; Amy J. Starr: None; Lisa F. Imundo: None; Andrew H. Eichenfield: None.

